# May-Thurner Syndrome: An Interesting Presentation of Recurrent, Unprovoked Deep Vein Thrombosis

**DOI:** 10.7759/cureus.43491

**Published:** 2023-08-14

**Authors:** Bethany A Phillips-Smith, Jazmin Jatana, Emma Carter, Sathyanarayana Machani, Dean J Wickel

**Affiliations:** 1 Family Medicine, West Virginia University School of Medicine, Wheeling, USA; 2 Vascular Surgery, West Virginia University School of Medicine, Wheeling, USA

**Keywords:** catheter-directed thrombolysis, thrombolysis, imaging to diagnose may-thurner syndrome, may-thurner's syndrome, hypercoagulability, deep vein thrombosis (dvt)

## Abstract

May-Thurner Syndrome (MTS) is a unique condition characterized by the compression of the left iliac vein by the right common iliac artery, which causes venous outflow obstruction and a high risk of venous sequelae. May-Thurner Syndrome is a condition that is primarily observed in females and is an uncommon cause of deep vein thrombosis (DVT). The more common presentation of DVT is in the lower left extremity, although there have been cases of right-sided formation. In this case report, we present a patient with unprovoked, recurrent, left-sided deep vein thrombosis in a 70-year-old woman. The aim of this case report is to highlight this uncommon condition and to suggest consideration of MTS in the setting of a patient with recurrent unprovoked DVTs of the same extremity.

## Introduction

May-Thurner Syndrome (MTS), which is also referred to as iliocaval venous compression syndrome or Cockett’s Syndrome, is a condition of compression in the iliocaval venous territory that leads to outflow obstruction [1]. The right common iliac artery passes in a normal anatomical course, over the proximal part of the left common iliac vein; however, May-Thurner anatomy is described as an abnormal compression of the left iliac vein by the right iliac artery against the fifth lumbar vertebrae. This anatomical variant has been shown to involve over 20% of the population, yet only a certain number of people will develop deep vein thrombosis, as there is believed to be a contribution from other factors [2,3]. This direct compression from the right common iliac artery also induces intimal proliferation in the left common iliac vein (LCIV) due to endothelial irritation from the pulsatile nature of the overlying artery [2,4,5]. These anatomic characteristics, in combination, lead to a decreased caliber of the vein. This condition, therefore, increases the risk of venous occlusion and venous hypertension. It is estimated that most individuals with May-Thurner anatomy are asymptomatic and may never present with acute DVT. In those that do become symptomatic, it is most commonly observed in females in the second to fourth decades of life [6-8]. It is estimated that MTS might account for about 2-5% of lower extremity venous disorders [9-11]. The classical presentation of MTS is the compression of the left common iliac vein between the fifth lumbar vertebrae and the right common iliac artery, increasing the risk for deep vein thrombosis in the left leg [4]. Though fewer in number, there have been cases of right-sided venous thromboembolism as well [4,12]. There are certain risk factors that are associated with MTS, which include the female sex, dehydration, hypercoagulable conditions such as pregnancy and postpartum, oral contraceptive use, severe scoliosis, surgery, and prior radiation [4,9,10,13].

Treatment modalities for acute DVTs include both anticoagulation and endovascular therapies. The majority of DVTs are able to be treated with anticoagulation. In patients with underlying MTS, however, there is an increased risk of recurrent thromboembolic events and venous hypertension. Our goal is to stress the importance of screening for this condition, so that patients with May-Thurner anatomy can be recognized, and intervention enacted, to decrease their risk of recurrent events.

The number of DVTs secondary to MTS is small compared to the total case number of total DVTs, though we feel that it is important to consider MTS as an etiology in the right clinical setting. As with any acute DVT, other etiologies must also be considered, such as provocation due to oral contraceptives or other external compression of the iliac vein due to pregnancy or pelvic mass. The purpose of this case presentation is to emphasize the necessity of considering MTS in the differential diagnosis of a patient with repeat unprovoked deep vein thrombosis in the same extremity.

## Case presentation

The patient is a 70-year-old Caucasian female with a past medical history of essential hypertension, hyperlipidemia, obstructive sleep apnea, prior cerebrovascular accident in 2017, multiple miscarriages, prior unprovoked left lower extremity deep vein thrombosis (2012 status post IVC placement and thrombolysis). Her family history included a clotting disorder in her biological mother.

The patient presented to the emergency department with left lower extremity edema of one-day duration. Upon reviewing the history of the present illness, she denied hormone replacement therapy, malignancy, recent surgery, recent travel, or periods of prolonged immobility. The patient’s Well’s score for DVT was three points due to leg swelling and previously documented DVT.

Physical exam was significant for tachycardia and hypertension with left lower extremity edema up to the level of the knee without warmth or erythema. There were no skin color changes and no sensory or motor deficits. Dorsal pedal pulses were 2+ bilaterally. Homans' sign was negative.

**Figure 1:**
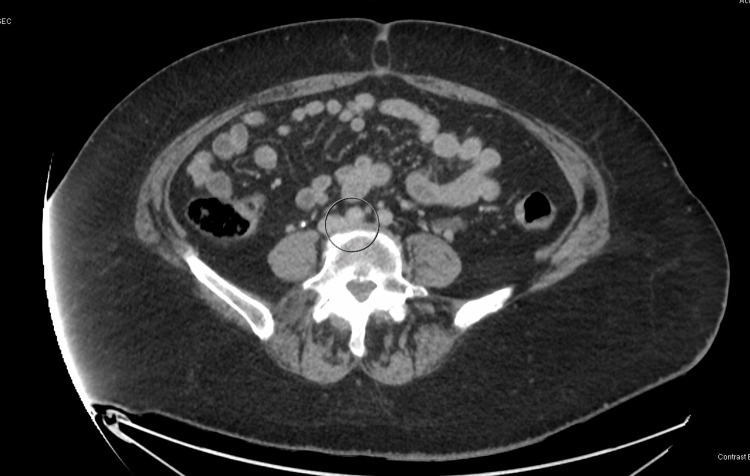
Compression of the left iliac vein by the right common iliac artery

Peripheral venous duplex revealed acute DVT in the left lower extremity, extending from the groin to the ankle. CT chest for pulmonary embolism with IV contrast was unremarkable. In addition, the thrombophilia panel was negative for beta-2 glycoprotein antibodies, anticardiolipin antibodies, Factor V Leiden mutation, and prothrombin mutation, with normal values for protein S and protein C activity, and lupus anticoagulant. An echocardiogram with bubble study was also performed to rule out paradoxical emboli and revealed a normal EF of 65% and no acute clots. The patient was subsequently started on a heparin drip and cardiothoracic vascular surgery was consulted. Vascular surgery initially opted for medical management with Eliquis. However, due to high suspicion of MTS, a CT venogram abdomen/pelvis was obtained (Figure 1). It revealed a significantly decreased caliber of the left common iliac vein with a mass effect, as it courses between the common iliac arteries and L4/L5 disc. These findings are conclusive for May-Thurner Syndrome.

**Figure 2:**
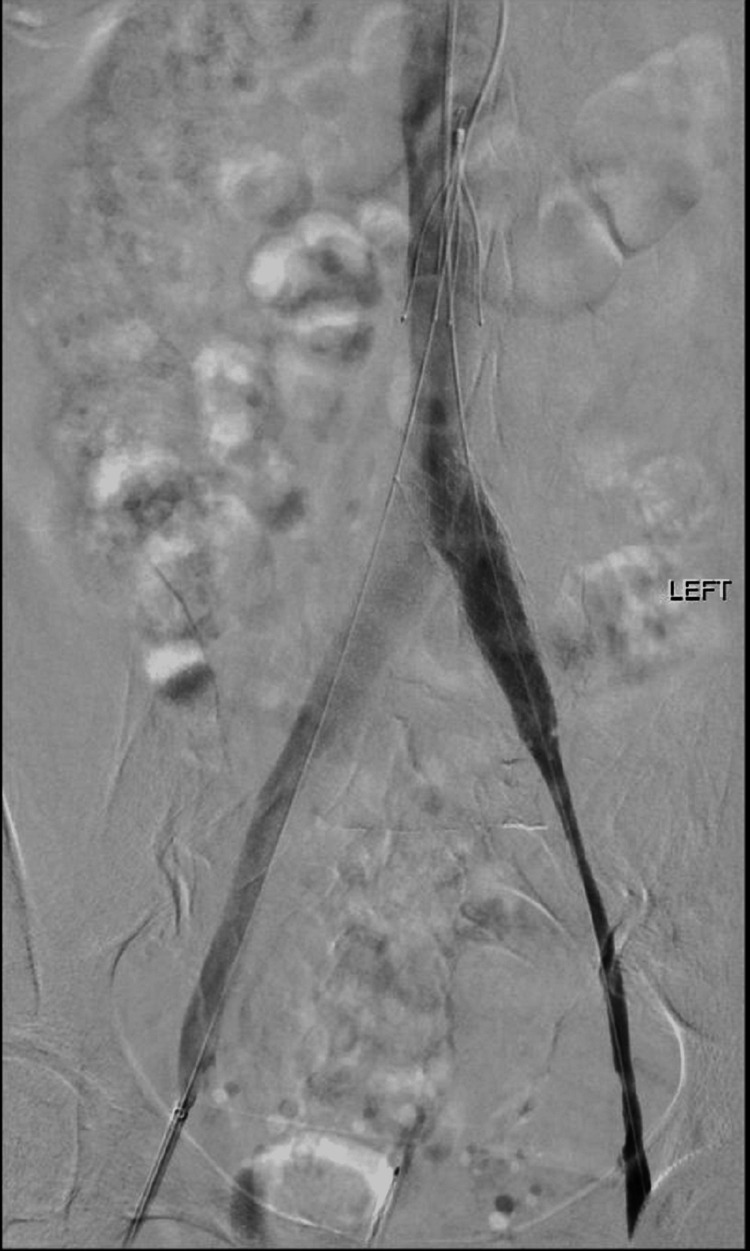
Left iliac vein angiography prior to stenting

**Figure 3:**
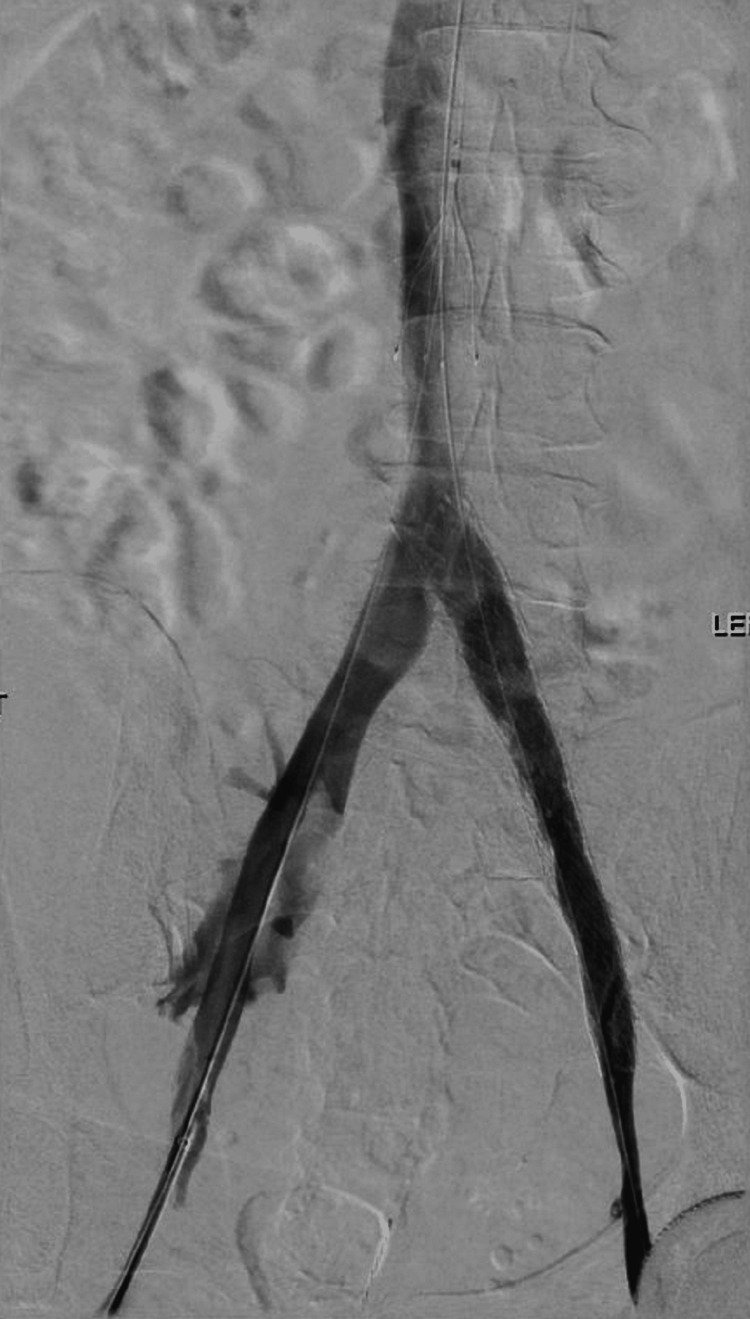
Left iliac vein angiography after stent deployment

Following the CT venogram findings, vascular surgery opted for IVC filter insertion followed by thrombolysis with venous stenting the following day (Figures 2, 3). Therapeutic Lovenox was initiated so as to bridge the patient to Warfarin as lifelong coagulation was also recommended. The patient tolerated these procedures well and was able to be discharged after a six-day admission. We discussed with her our interest in publishing this case, and the patient was willing and consented verbally and in writing.

## Discussion

This case presentation discusses the occurrence of recurrent DVTs in a patient with no apparent risk factors. Despite the recognition of May-Thurner Syndrome dating back to the mid-1900s, it remains an uncommon condition with limited available literature. Our goal is to reinforce the importance of suspecting May-Thurner Syndrome in all patients with an acute recurrent DVT in the same lower extremity, especially in those patients that meet certain known risk factors such as female sex or left lower extremity involvement.

A common presentation for this condition is venous insufficiency with episodic leg swelling, varicose veins, venous ulcers, or venous stasis dermatitis, but sometimes, a lower extremity DVT with painful swelling may be the first presenting symptom [4]. According to a systematic review by Kaltenmeier et. al., it was determined that 52.4% of patients presented with a lower extremity DVT prior to the diagnosis of MTS, which further reinforces the importance to consider this diagnosis in cases of acute DVT. This systematic review also determined that in the subset of patients that are being evaluated for chronic venous insufficiency (CVI), 2% to 5% will be diagnosed with May-Thurner Syndrome, and the average age at presentation is 42.6 ± 16.9 [6]. With MTS being associated with deep vein thrombosis, and DVTs known to cause complications such as pulmonary emboli, it is imperative to determine the underlying etiology to prevent recurrence. In regards to our patient, had we not considered and evaluated for MTS during this admission, she could have had a delayed diagnosis and potentially further complications.

The diagnosis of MTS is important to discuss and begins with a thorough history and physical exam, exploring the possibility of a provoked DVT, assessing for symptoms of chronic venous stasis, and exploring family history. Initial testing often begins with duplex ultrasound to confirm the presence of DVT. This option is ideal for assessing the lower extremity, however, visualization of clot burden in the pelvic region may be difficult to assess via this method [14]. Therefore, cross-sectional imaging, such as CT venography, MR venogram, or intravascular ultrasound (IVUS), should be utilized, as they have higher combined sensitivity and specificity for establishing the diagnosis of MTS. These three diagnostic modalities are excellent options for diagnosing this condition, and the choice between them may vary by provider or hospital, as no standard exists for recommending one over another [15]. IVUS has recently been increasing in popularity for establishing the diagnosis, as it has the inherent benefit of showing dynamic compression of the internal iliac vein by the overlying artery and can aid in localizing intimal lesions. Also of note, an important consideration, as discussed by Poyyamoli et al., is to hydrate the patient appropriately prior to imaging, as hypovolemia may negatively affect the specificity of diagnosis [4].

In regards to our patient, we opted for CT venography as our diagnostic modality due to the higher sensitivity and specificity compared to ultrasound, and the fact that our facility does not have access to IVUS. The CT venogram concluded the presence of MTS with the diminished caliber of the left common iliac vein between the L4-5 disc and the proximal common iliac artery. The diameter of the left common iliac vein was successfully evaluated in the sagittal view on the CT scan and was found to be significantly diminished. A study by Carr et. al. explored the correlation of the diameter of the LCIV with the risk of developing DVT in the lower extremities. They determined that patients with DVT had an average LCIV diameter of 4.0 mm, and those of the control group had an average of 6.5 mm, which was determined to be a six-fold increased risk of DVT formation [16].

Intervention for patients with confirmed May-Thurner anatomy can be both conservative and surgical, with the end goal being improved venous outflow. Patients who are mildly symptomatic without a DVT present can be managed conservatively with compression stockings and monitoring. In patients with moderate to severe symptoms in the absence of DVT, the goal is to reduce recurrence and venous sequela. In those patients with May-Thurner anatomy and acute DVT, the mainstay of management involves lessening the clot burden and improving venous outflow with endovascular interventions [4]. Surgical intervention has advanced from open techniques to more modern endovascular therapy with angioplasty and stenting, followed by anticoagulation. In eligible patients, catheter-directed thrombolysis or mechanical thrombectomy are excellent options to remove the thrombus burden, visualize the affected area, and stent the stenotic lesion [17-19]. Lifelong anticoagulation is often recommended after surgical intervention, though there is some debate. There are studies that suggest that anticoagulation be used for a shorter period of time, between six and 12 months since the underlying anatomical abnormality has been addressed and corrected [20].

Re-thrombosis after stent placement is uncommon but does occur. In the event of re-thrombosis, the need for re-intervention is minimal. According to one study, severe in-stent restenosis, defined as less than 50%, occurred in 5% of stented limbs at 72 months, with thrombophilia by itself not being a risk factor. They noted that the largest contributor to re-stenosis was actually the underlying extent of thrombotic disease [19]. It can be deduced that those with May-Thurner Syndrome plus underlying atherosclerosis may be at a higher risk of needing repeat intervention.

In the case of our patient, our vascular surgeon opted for a multipart treatment beginning with the placement of an IVC filter, followed by thrombolysis via a Craig McNamara catheter, with an infusion of tissue plasminogen activator (tPA) at 1.5 mg/hr. The following day, our patient was taken back to the operating room for further intervention with venous angioplasty and stenting. Our patient tolerated the procedure well and was discharged on anticoagulation with warfarin, as a direct oral anticoagulant was not affordable. An endovascular approach with angioplasty and stenting is an excellent choice for the treatment of May-Thurner Syndrome, as it corrects for the underlying anatomy and thus the etiology of the related symptoms of CVI and recurrent DVT.

## Conclusions

Given the rarity of the diagnosis of MTS in cases of acute occlusion, this case report serves to increase awareness of this important disorder. As discussed in this particular case, invasive intervention is crucial to reduce the occurrence of secondary complications such as lower limb venous hypertension and recurrent venous occlusion. This patient's case is unique in the fact that our patient presented with a recurrent deep vein thrombosis in the left lower extremity, 10 years apart. Being 70 years old, she is also outside of the window in which patients commonly present. Looking forward, this patient will need repeated coagulation studies for a more complete evaluation. While she had a coagulation panel done immediately after her first DVT, the results were inconclusive. At that time, her protein C and protein S function were diminished, however, she was actively on warfarin therapy. As warfarin can cause falsely low levels of protein C and S activity, it is difficult to determine if the patient has a true deficiency based on these prior results.

This case is a clinical scenario of the necessity for surgical intervention despite minimal symptoms at onset. The standard treatment for our patient, given the initial evaluation, would have been outpatient anticoagulation. Had we not considered MTS as a possible etiology, she may have had a different outcome. Thus, we feel it is important to always consider MTS as a possible etiology in the correct clinical scenario. If May-Thurner Syndrome is diagnosed early, followed by appropriate clinical intervention, it can significantly reduce adverse outcomes and reduce morbidity and mortality in the affected patients.
